# Bone combined cement grafting in giant cell tumor around the knee reduces mechanical failure

**DOI:** 10.1007/s00264-018-3939-2

**Published:** 2018-04-27

**Authors:** Wangsiyuan Teng, Peng Lin, Yong Li, Xiaobo Yan, Hengyuan Li, Binghao Li, Zhan Wang, Yan Wu, Shengdong Wang, Xingzhi Zhou, Zenan Wang, Zhaoming Ye

**Affiliations:** 1 Department of Orthopedics, Second Affiliated Hospital of Zhejiang University School of Medicine/Orthopedics Research Institute of Zhejiang University, Hangzhou, Zhejiang Province People’s Republic of China; 2 Department of Orthopedics, Second Affiliated Hospital of Zhejiang University School of Medicine, No.1511, Jianghong Road, Hangzhou, 310000 China

**Keywords:** Distal femur, Giant cell tumor, Proximal tibia

## Abstract

**Objectives:**

The aims of our study are (1) to explore the risk factors of mechanical failure (MF), (2) to figure out an index to evaluate this risk, and (3) to select an optimal reconstruction strategy to reduce this risk.

**Methods:**

We retrospectively reviewed 104 patients from Dec. 2008 to Mar. 2016, undergone extensive knee curettages in our institution. Radiographs and post-operative interviews were used to classified cases of MF. Relative factors (age, tumor location, the invaded area, etc.) were also collected and analyzed by SPSS software.

**Results:**

Thick subchondral bony layer (*p* = 0.006) and combined grafting of the cement and bone (*p* = 0.006) had lower risk of mechanical failure. Mechanical failure appeared to happen in the femur (*p* = 0.012) more easily. The ROC curve (AUC = 0.722) reveals that less post-operative bony layer (≤ 3.3 mm) is more likely to cause mechanical failure. The Kaplan-Meier survival curve showing increased survival in those patients after a combination grafting surgery (HR, 3.799; *p* = 0.006).

**Conclusion:**

Based on our study results, combined grafting of the cement and bone reduced the risk of mechanical failure in the knee due to the thin subchondral bone layer (SCB), especially in the femur.

## Introduction

Giant cell tumor of the bone is a local aggressive benign bone tumour, and it has a potential for metastases [1]. It is commonly located in the epiphyseal regions of long bones, such as the distal femur and proximal tibia. Because of its incidence peaks in the third and fourth decade [2], preservation of the joint is of importance for these young patients [3]. And intralesional extensive curettage becomes the mainstay of treatment for primary giant cell tumour of the bone nowadays [1]. To make a complete tumour removal, the structure of the joint surface may be jeopardized during the curettage procedure around the knee, which subsequently leads to mechanical failure. This post-operative mechanical failure is described as irregularity, deformity, fracture, or even collapse of articular surface, resulting in discomfort or poor function of the knee.

To date, several studies indicated that less residual subchondral bone and larger area of the subchondral bone invaded by GCT were more likely to accelerate the procedure of degenerative changes in the bone [4, 5]. Supplemental bone grafting in the subchondral bone area might reduce the degenerative development [6, 7]. Considering that destruction of the articular cartilage and subchondral bone due to extensive curettage led to occurrence of mechanical failure; it was crucial to figure out reliable clinical measurements to predict and reduce this risk.

We hypothesized that less residual subchondral bone and larger area affected by the tumor tended to induce the degenerative changes around the knee. And the degenerative changes resulted in mechanical failure. Therefore, we attempted to answer the following questions in our study: (1) Whether there were any significant differences in residual subchondral bony layer, the area of the subchondral bone invaded by the tumour, Campanacci grade, surgical options, or other factors between the mechanical failure group and the control one. (2) Whether we could use the residual subchondral bony layer to well evaluate the risk of mechanical failure. (3) Whether supplement grafting could reduce this risk.

## Materials and methods

**Fig. 1:**
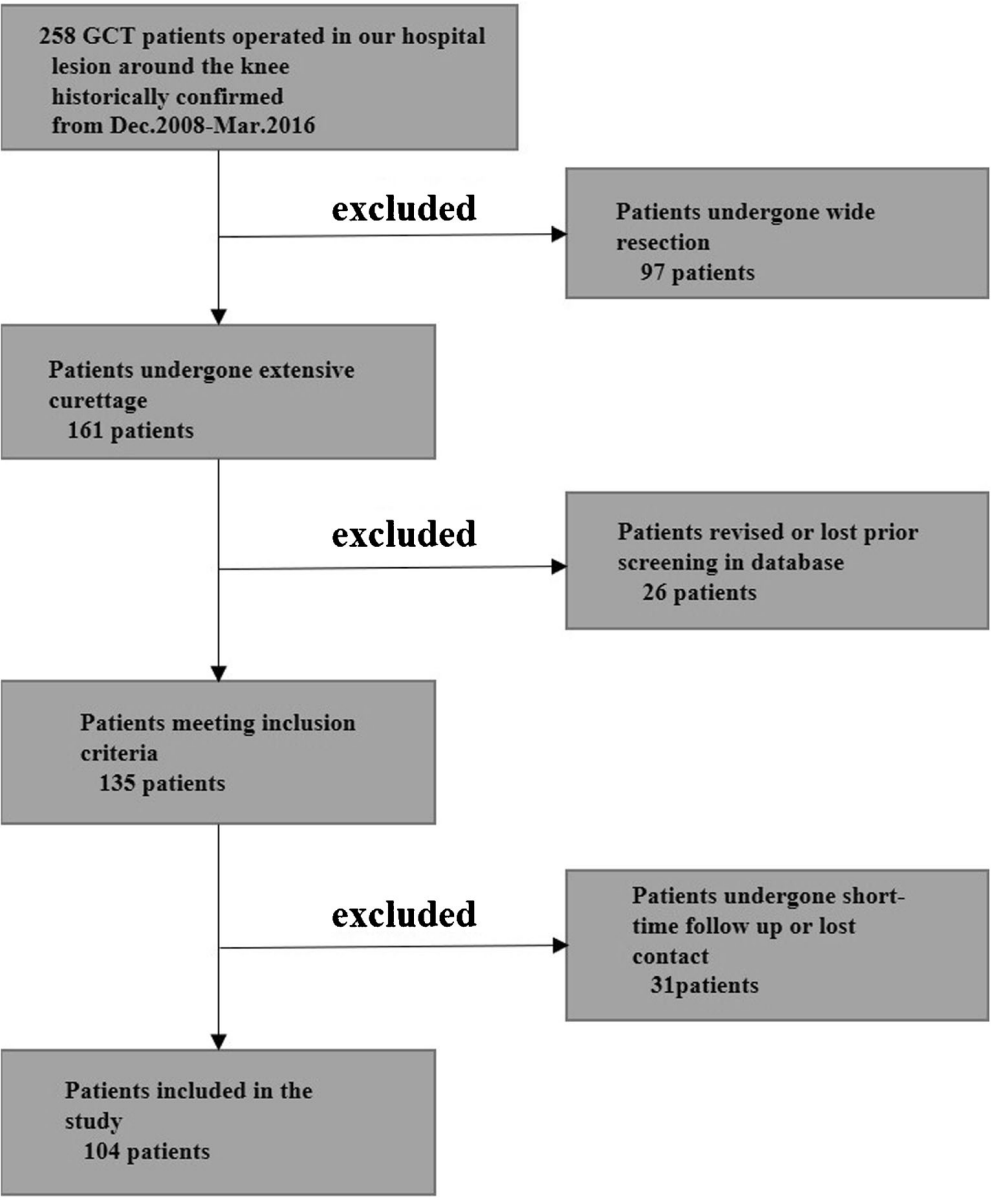
Flow chart of the study

This was a retrospective study based on patients’ records and imaging data. Two hundred fifty-eight patients with a giant cell tumor in the knee were treated in our institution from Dec. 2008 to Mar. 2016. In this cohort, we excluded 97 patients treated with wide excision primarily, 26 patients with incomplete of information, and 31 patients of less than 12 months’ follow-up or loss of contact. The remaining 104 patients met the criteria which included primary surgical procedure of extensive curettage done in our institution and a minimum duration of 12 months follow-up. The detail flow chart of the study was shown in Fig. 1. All operations were performed by four experienced surgeons. And the operative procedures were similar. Two types reconstruction were performed: cement packing alone and cement-combined bone grafting.

### Surgical technique

CT scan of the lesion helps direct a lateral or medial approach to access the more affected side. Generally, the cortical window was made at first. Part of the affected soft tissue capsule over the tumour cortex should be removed. All primary extensive curettage followed with high-speed burr and electrocauterization repeatedly to completely remove the tumour. After curettage, the cavity was rinsed with phenol, hydrogen peroxide solution, distilled water pulse irrigation, and then filled with cement alone or cement-combined bone grafting (autograft or allograft bone was implanted between the cement and subchondral bone). Fifty-seven patients underwent cement packing alone and 47 patients underwent cement combined bone grafting. For every patient in this cohort, the post-operative functional exercises were almost identical.

### Patient evaluation

All the medical records and imaging data were reviewed. The most recent post-operative radiographs were selected to make the evaluation.

**Fig. 2:**
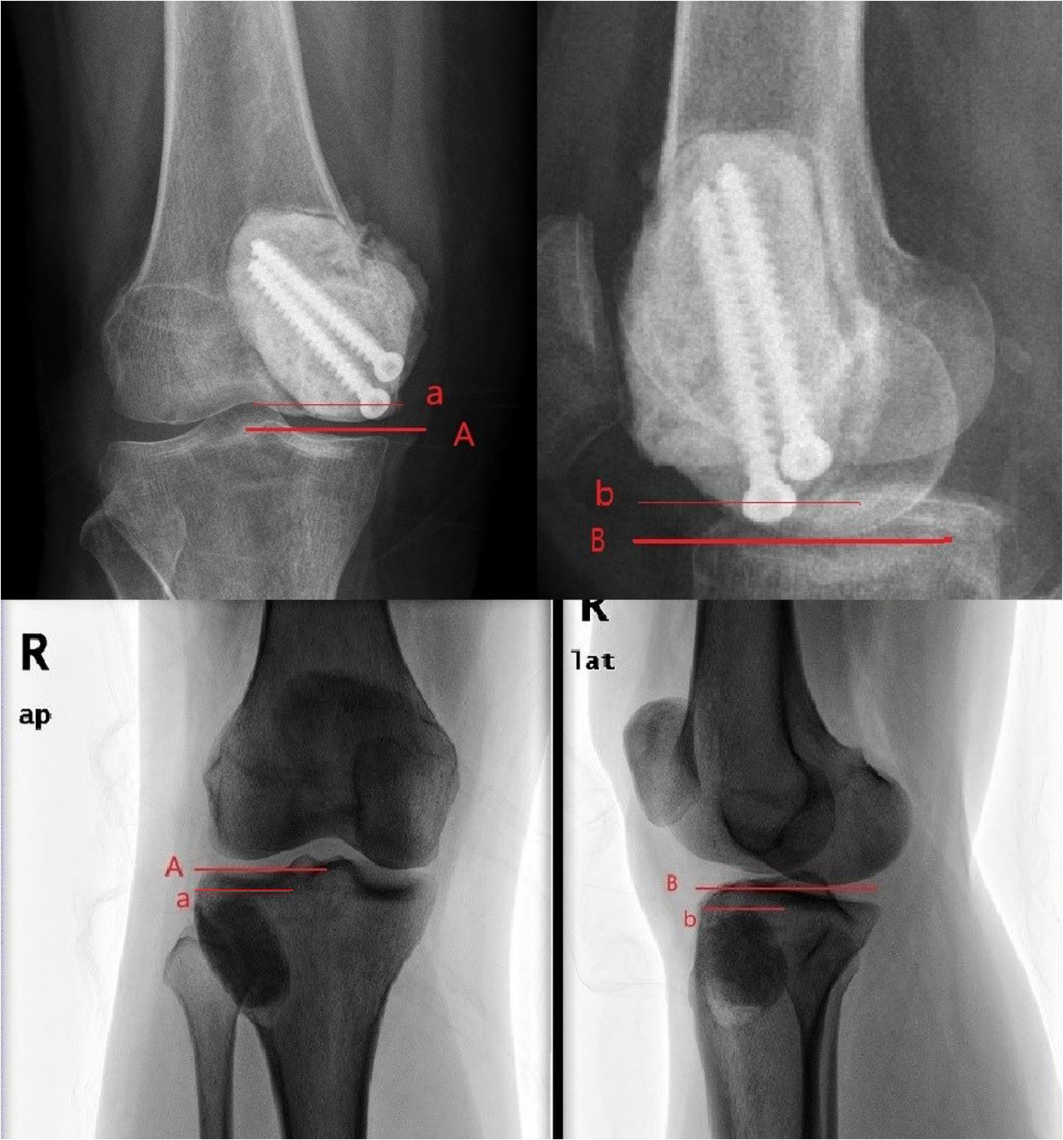
Method used to measure the area of the subchondral bone affected by the giant cell tumour of the bone: 1. Width of cavity filled by cement in the anteroposterior plane (a) and the lateral plane (b). 2. Width of corresponding compartment (epicondyle to middle of joint) in the anteroposterior plane (A) and the lateral plane (B). Width of corresponding compartment in the anteroposterior plane would be replaced by width of whole articular surface when the lesion invaded almost the entire surface. 3. (a × b/A × B) × 100 = % of the subchondral bone area affected by tumour

The following measurements were made on each radiograph:

1. We defined the mechanical failure happened, when the patient met any of the following conditions:
  - presented grade 2 or more radiographic performance on plain film according to Aboulafia’s radiographic classification [5] (Table 1);
  - had at least one micro-fracture presence on CT scan;
  - complained a persistent pain of the knee six months after the surgery and had mild knee degeneration without any signal of local recurrence
2. The area of the subchondral bone invaded by GCT as described by Chen [8] (Fig. 2).
3. The shortest distance from the articular surface to the nearest margin of the cement on radiographs was defined as the thickness of the residual subchondral bone layer. This distance was measured by ITK-SNAP software version 3.6.0.

**Table 1:** Radiographic evaluation scale of Albert J. Aboulafia

Grade 0	Anatomic articular contour without joint space narrowing
Grade 1	Minimal irregularity of articular surface
Grade 2	Moderate irregularity of articular surface with joint space narrowing < 2 mm or minimal (< 5°) varus/valgus deformity
Grade 3	Deformity of articular surface with > 2 mm joint space narrowing or evidence of subluxation
Grade 4	Collapse of articular surface with deformity (> 5°) varus/valgus, subluxation > 5 mm or loss of articular surface with resultant bone on the bone appearance

Two members in our team (T.W. and L.P.) performed the measurement independently. Every radiograph of GCT patients was measured three times by each member to reduce the dating error.

Routine follow-up with radiographs every three months for the first year was done in all patients, then every six months for two or three years, and then annually thereafter.

Hospital for Special Surgery knee-rating score was used for evaluating functional outcome status post-operatively [9].

### Statistical analysis and study size

Factors related to mechanical failure, such as age, gender, tumour location, Campanacci grade, area affected by tumour, the residual subchondral bone layer, and the reconstruction types of primary curettage were analyzed by multivariate analysis with the COX proportional hazard model. For HSS score, nonparametric test was used. Then, we made a receiver operating characteristic (ROC) curve to assess the efficacy between the residual subchondral bone layer and the risk of post-operative mechanical failure. Based on the curve, its Youden index was found. A Kaplan-Meier survival estimator was made to evaluate the failure risk after different surgical options. All the statistical analyses were processed by SPSS software version 24.0.

### Demographics and description of study population

The mean age of the first diagnosis was about 35 years (SD, 11.2; range, 17–70) and the follow-up time was a mean duration of 33 months (SD, 18.77; range, 12–95). There were 58 females and 46 males. Sixty-three cases located in the distal femur and 41 in the proximal tibia. Thirty-two cases suffered mechanical failure. Eleven patients developed a local recurrence, and two patients who were treated with wedge resection of lung or pulmonary lobectomy were found with pulmonary metastasis.

## Results

Thirty-two mechanical failure cases were found this time. According to the multivariate analysis of the COX proportional hazard model (Table 2), tumour locating on the tibia (*p* = 0.012), thick subchondral bony layer (*p* = 0.006), and combined grafting of the cement and bone (*p* = 0.006) had lower risk of mechanical failure. The results revealed that the risk of post-operative mechanical failure on the femur could be three times higher as that of on the tibia. Comparing with the combination grafting, the reconstruction of cement packing alone had almost four times the risk of mechanical failure.

**Table 2:** Possible factors at multivariate analysis with the COX proportional hazard model

			B	SE	*p* value	HR	95.0% CI of HR	
							Lower	Upper
Gender (female = 0, male = 1)	Female	58	− 0.029	0.464	0.951	0.972	0.391	2.415
	Male	46						
Age (≤ 30 = 0, > 30 = 1)	≤ 30 years	37	0.003	0.491	0.995	1.003	0.383	2.626
	> 30 years	67						
Medial/lateral^*^	Lateral	39	− 0.237	0.402	0.556	0.789	0.359	1.735
	Medial	54						
Femur/tibia (femur = 0, tibia = 1)	Femur	63	− 1.253	0.498	0.012	0.286	0.108	0.757
	Tibia	41						
Post-subchondral bone thickness			− 2.605	0.949	0.006	0.074	0.012	0.474
Area affected by tumor			0.008	0.013	0.545	1.008	0.983	1.034
Campanacci grading	1	34	0.385	0.344	0.264	1.469	0.748	2.885
	2	55						
	3	15						
Reconstruction types (cement + bone graft = 0, cement alone = 1)	Cement + bone graft	47	1.335	0.483	0.006	3.799	1.475	9.787
	Cement alone	57						

*Eleven patients’ lesions invaded almost the whole articular surface

**Fig. 3:**
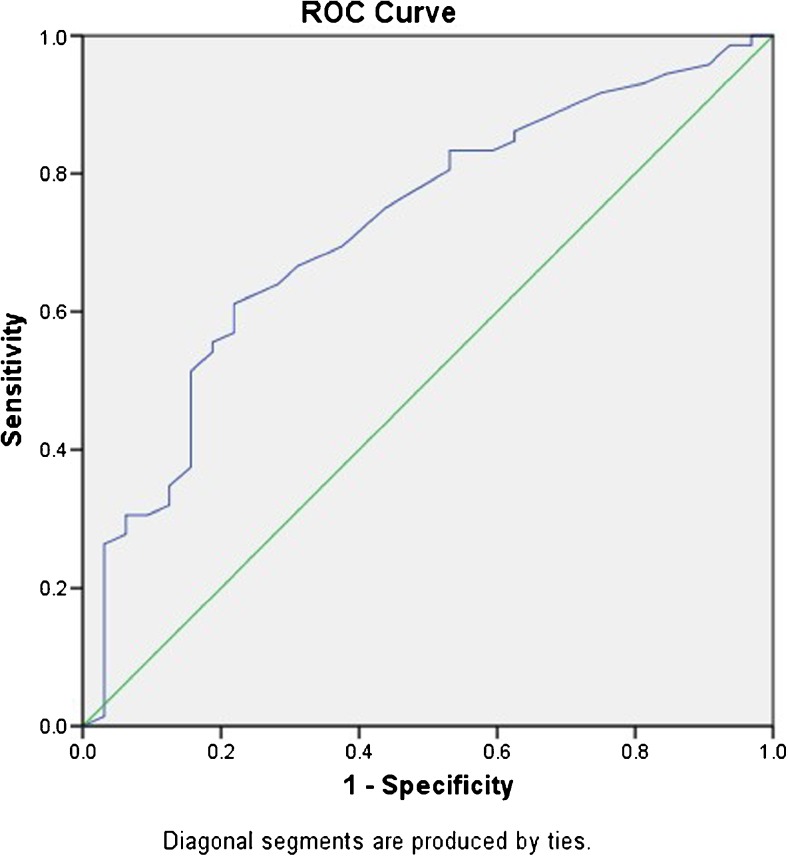
The ROC curve showing increased risk of mechanical failure in patient of the thin subchondral bony layer after the surgery (AUC = 0.722, its 95% CI is from 0.617 to 0.827)

The ROC curve (Fig. 3) showed that evaluating the risk by measuring residual thickness of subchondral bony layer was reasonable (AUC = 0.722, its 95% CI is from 0.617 to 0.827). Mechanical failure happened more easily in thin post-operative bony layer (≤ 3.3 mm).

**Fig. 4:**
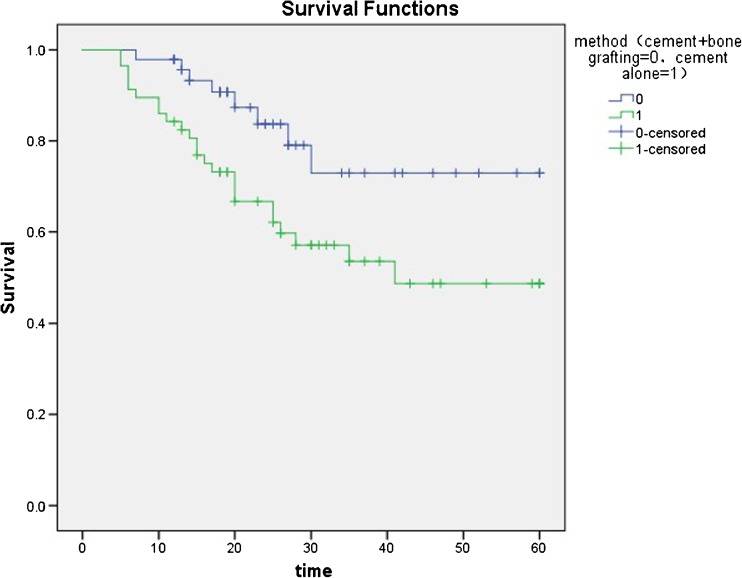
Kaplan-Meier survival curve showing increased MF-free survival in patients after a cement + bone grafting surgery (HR, 3.799; *p* = 0.006)

Cement-combined bone grafting had a better outcome in MF-free survive. The Kaplan-Meier survival curve (Fig. 4) showing increased MF-free survival in patients after a cement combined bone grafting surgery (HR, 3.799; *p* = 0.006).

## Discussion

Though denosumab was used in GCT treatment [10], extensive curettage was still the mainstay of management for primary giant cell tumour of the bone [1, 2, 4, 6]. To eliminate the GCT cells completely, we routinely enlarged the residual cavity with a high-speed burr at least 3~5 mm, except for the articular cartilage aspect. On the articular side, only the most affected area (not all the subchondral bone) was removed, if the subchondral bone layer was less than 1 cm. This procedure had inevitable damages for the articular cartilage [2] or subchondral bone. Thus, the capacity of pressure dispersal decreased due to defect of the subchondral bone layer. Adapting to this load alteration, the articular surface initiated the bone remodeling leading to degeneration of the bone and change of mechanical properties. The aim of our study counseled patients on indicating their possibility of mechanical properties occurrence for the principal treatment options.

### The subchondral bone

The GCT patients had a higher post-operative risk of degeneration on the knee. A research carried by [11] showed that the subchondral bone joined joint degeneration and had functional interactions between the bone and cartilage. The thickness of the subchondral bone appeared to contribute to this progression in Campanacci’s study [12]. In Goldring’s study [13], the subchondral bone layer mechanics deformation might have something to do with degeneration of the knee joint. Some researches indicated that the subchondral bone layer had a shock-absorbing effect after the surgery, and joined the remodeling procedure later [4, 14, 15].

By reviewing these studies above, we supposed that the damages of the articular subchondral bone would lead to mechanical failure after curettage and enough post-operative thickness of residual bony layer appeared to reduce the risk. In other words, it is reasonable to use the residual thickness of the subchondral bone post-operatively to indicate the risk of mechanical failure.

Chen and his colleagues [8] defined that the subchondral bone was destructed if its thickness was less than 3 mm, and found that the greater the lesion area, the worse the joint function. Abdelrahman [16] demonstrated that the probability of degeneration (in the group where the thickness was less than 10 mm) was 2.5 times that of the group where the thickness was more than 10 mm. To some extent, our study reinforced those conclusions: the less subchondral bony layer remained, the higher the risk. To achieve a complete tumour curettage, more tissue was removed during the surgery, leading to more damage on the subchondral bone layer. This resulted in an irregularity of the joint surface or instability of the lower extremity, possibly leading to progression of mechanical failure around the knee.

In our current study, all cases were strictly evaluated based on the radiographic evaluation scale, and 32 cases of mechanical reconstruction failure were found during a 33-month follow-up. The results showed that less subchondral bony layer was more dangerous (*p* = 0.006). With the data available, we then made its ROC curve and found its Youden index (SCB = 3.3 mm). This point was very close to Chen’s assumption [8] and was supported by other surveys [2, 4]. It implied that if the subchondral bone layer was less than 3.3 mm, patients had a greater chance of mechanical failure post-operatively.

### Reconstruction option

**Fig. 5:**
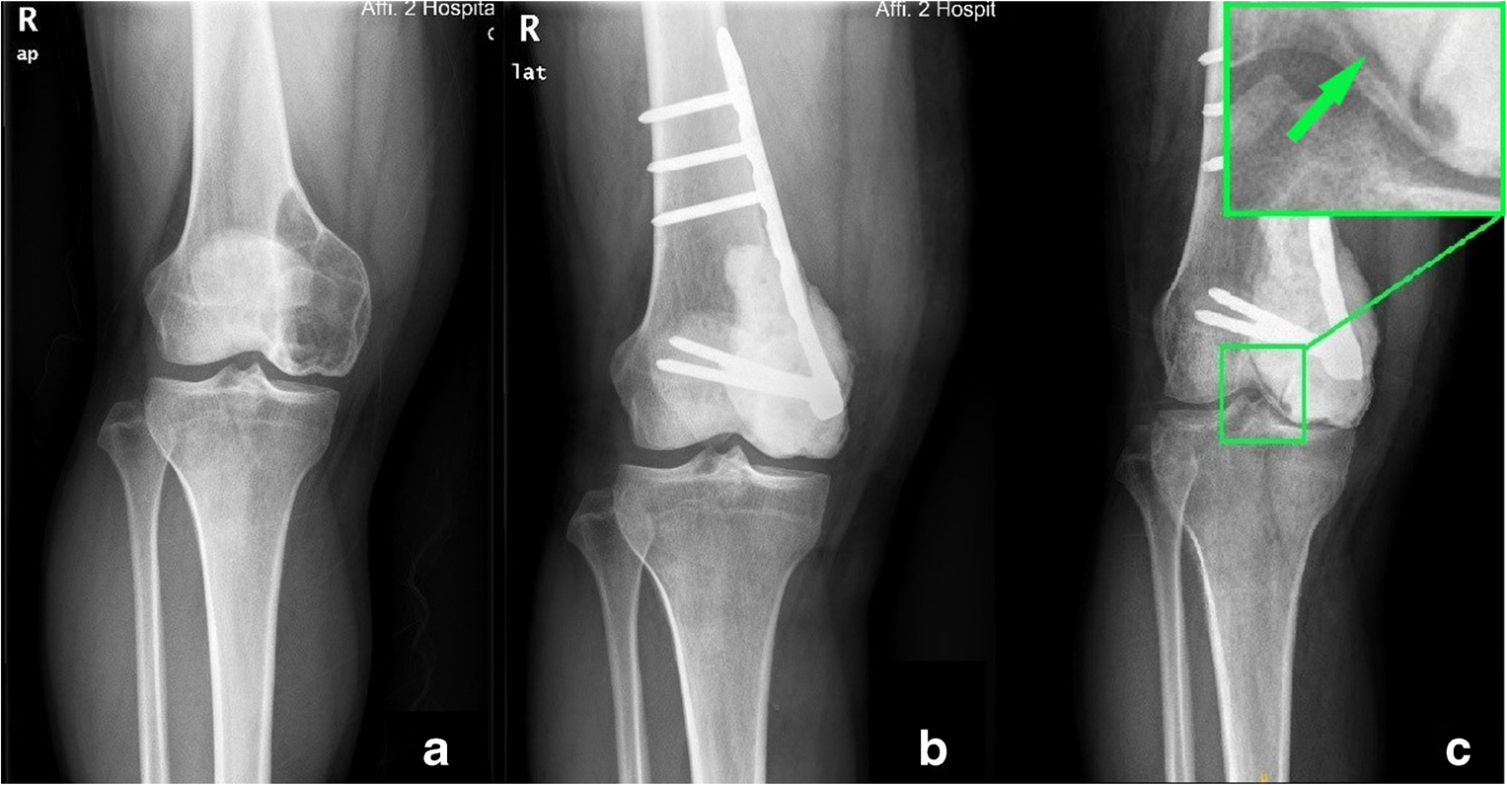
**a** AP view of a 33-year-old male patient with GCT. **b** Extensive curettage, cement filling was performed. This AP showed the knee 2 days following the surgery. **c** A sclerotic rim occurred (green box), separating the cement from the surrounding bone and subchondral bone layer. The artificial surface collapsed

Though cement packing was regarded as a good reconstruction method in some researches [17, 18], its disadvantages were that cement was non-biodegradable and had no ability to grow biologically into the surrounding host bone. Consequently, a sclerotic rim occurred, separating the cement from the surrounding bone and subchondral bone layer [19]. Welch et al. [20] described this rim could decrease the shock-absorbing capacity of the subchondral bone layer. When walking, prosthesis loosening, and slight rolling happened due to the separation around the cement. Then the articular cartilage and subchondral bone were damaged, which resulted in mechanical failure (Fig. 5). Based on the results, the option of reconstruction in curettage made a significant difference in mechanical properties. The method of cement packing alone had poor biomechanics (HR, 3.799; *p* = 0.006).

The bone graft was the mechanical support for subchondral defects [21, 22]. But it failed to reconstruct the lesion alone for its weak strength when packing alone. According to Szalay’s results [14], there existed an increased risk of degenerative changes during the first 24 months post-operatively in joints of weight-bearing bones reconstructed with the bone grafting alone. Animal model experiments [21] proved that the strength of subchondral defect filled with the cancellous bone was only slightly greater than that of empty cavity. This implies that, the bone grating alone could lead to collapse and fracture of the subchondral bone easily.

Some researches indicated that the bone grafting in subchondral zone had a shock-absorbing effect after the surgery, and joined the remodeling procedure later [4, 14, 15]. Benevenia [6] found that the bone grafting in the subchondral bone area reduced nononcologic complications in patients of giant cell tumour of the bone. In combination grafting, the cement packing gave a strong supplement to avoid the joint mechanical deformation. And the bone graft provided a good bone conduction. This process of the bone conduction was mentioned clearly by Yano’s team in their study of mice model [23]. They discovered that the labeled osteoblasts derived from the grafted bone gradually decreased at day seven and completely disappeared at day 42. The new formed bone completely consisted of the other labeled osteoblasts derived from the host. This experiment indicated that the cells contained in the grafted bone were gradually resorbed and replaced by the host cell, facilitating new bone formation.

According to our results, patients of combination grafting had lower incidence of mechanical failure (HR, 3.799; *p* = 0.006). We also discovered that this option had the capacity of being resorbed and replaced by the host bone in the long term and reconstructed the mechanical integrity of the bone. Thus, we recommended combination grafting of the cement and bone in extensive curettage as the optimal reconstruction strategy.

### Higher risk on the femur

Additionally, we found that tumour locating on the femur had higher risk of post-operative mechanical failure (*p* = 0.012). The results showed the risk on the femur could be three or more times high as that of on the tibia. It was assumed to be correlated with their roles in the motion of the knee joint. During the knee movement of flexion and extension, most of the relative motion between the femur and tibia was sliding. When it came to the last 20° in extension, the relative motion changed into rotation, resulting in an internal rotation of the femur. Similarly, an external rotation happened in the femur at the first 20° of flexion. This rotational force could be the main cause to induce direct destruction of the femoral condyles. Moreover, the meniscus located between the femoral condyle and tibia platform acted like a buffer. It protected the tibia platform by absorbing the pressure transferred down, especially in flexion and extension.

### Limitations

This study had some unavoidable limitations. It is retrospective and has a limited sample size. With larger numbers, some of the expected differences in groups of tumour-affected area might have become apparent. Though the Campanacci grade showed no significant difference between groups in our study, there existed selection bias that patients with lower-grade disease were more likely to be treated with curettage than those with higher-grade disease.

## Conclusion

In the light of our results, postoperative thickness of subchondral bony layer can be used to predict the risk of mechanical failure. Less subchondral bony layer and tumour on femur may be more dangerous. We recommended an extensive curettage with combined grafting of the cement and bone. This reconstruction option reduced the risk of mechanical failure in the knee when less subchondral bone layer remained, especially on the femur.
